# Inflammatory stays inflammatory: a subgroup of systemic sclerosis characterized by high morbidity and inflammatory resistance to cyclophosphamide

**DOI:** 10.1186/s13075-019-2057-x

**Published:** 2019-12-02

**Authors:** Aleksey Mitev, Lisa Christ, Daria Feldmann, Moritz Binder, Kim Möller, Anna-Maria Kanne, Thomas Hügle, Peter M. Villiger, Reinhard E. Voll, Stephanie Finzel, Florian Kollert

**Affiliations:** 1 Department of Rheumatology and Clinical Immunology, Faculty of Medicine, Medical Center University of Freiburg, University of Freiburg, Freiburg, Germany; 2 Department of Internal Medicine, Mayo Clinic, Rochester, MN USA; 3 Rheumatology Research Group, Institute of Inflammation and Ageing, University of Birmingham, Birmingham, UK; 4 Rheumatology, University Hospital Lausanne (CHUV), Lausanne, Switzerland; 5 Department of Rheumatology, Immunology, and Allergology, Inselspital, University Hospital Bern, Bern, Switzerland

**Keywords:** Systemic sclerosis, C-reactive protein, Cardiovascular disease, Cyclophosphamide, Cardiac arrhythmia, Inflammation

## Abstract

**Background/purpose:**

Elevated levels of C-reactive protein (CRP) in systemic sclerosis (SSc) have been linked to early inflammatory stages of the disease. This study has been designed to investigate CRP levels longitudinally in a cohort of SSc patients and to correlate these findings with comorbidities and disease characteristics.

**Methods:**

In this retrospective study, patients with SSc treated at the outpatient clinic of the Department of Rheumatology and Clinical Immunology, University Medical Center Freiburg, were analyzed. Only patients with at least three consecutive visits and at least 1 year follow-up were included in this study. CRP serum levels were measured at every visit and categorized as positive if CRP concentrations were ≥ 5 mg/l. Subjects with elevated CRP levels at more than 80% of visits were defined as inflammatory SSc. The longitudinal CRP profiles were correlated with disease characteristics and comorbidities.

**Results:**

A total of 1815 consecutive visits of 131 SSc patients were analyzed. Over the observed time span (7.6 (1.0–19.5) years), 18.3% (*n* = 24) of patients had continuously elevated CRP levels (inflammatory SSc), whereas in 29% (*n* = 38), CRP levels were always in the normal range. There was no association between disease duration and CRP levels at first visit. Inflammatory SSc was associated with male gender (*p* = 0.022), anti-Scl-70 antibodies (*p* = 0.009), diffuse cutaneous SSc (*p* = 0.036), pulmonary fibrosis (*p* < 0.001), rheumatoid arthritis (*p* = 0.007), and cardiac arrhythmia (*p* = 0.048). Moreover, patients with inflammatory SSc revealed higher modified Rodnan skin scores (*p* < 0.001); lower forced vital capacity (FVC) (*p* < 0.001), total lung capacity (*p* = 0.001), and diffusing capacity (*p* = 0.008); and faster decline of FVC per year (*p* = 0.007). Even treatment with cyclophosphamide (CYC) did not decrease CRP levels (*p* = 0.754).

**Conclusion:**

Inflammatory SSc is characterized by a more severe phenotype, high morbidity, and a large proportion of male patients. Even treatment with CYC does not alter CRP levels in this subpopulation with a high unmet medical need.

## Background

Systemic sclerosis (SSc) is a heterogenous disease characterized by vasculopathy, skin thickening, and internal organ involvement, such as interstitial lung disease (ILD) or renal crisis. Pathogenesis involves autoantibodies, endothelial cell and fibroblast dysfunction, and deposition of extracellular matrix, with the consequence of vasculopathy and fibrosis [1].

Treatment, which includes immunosuppressive drugs like cyclophosphamide (CYC) and even autologous hematopoietic stem cell transplantation (HSCT), remains challenging. Particularly, selection for HSCT is demanding; patients with an extensive disease like severe pulmonary fibrosis or cardiac involvement are prone to side effects, and studies have shown 6–10% treatment-associated mortality [2, 3]. In contrast, patients without severe organ involvement might be stable over years without the need for systemic therapy. Hence, prognostic markers to guide treatment decisions are needed.

The pathogenesis of SSc is thought to involve an initial inflammatory phase, characterized by a Th1- and Th17-immune response, which is followed by a switch to Th2, leading to irreversible fibrosis and a “burn-out” of the disease [4]. The early inflammatory phase is considered to be promising for therapeutic interventions, in contrast to non-inflammatory phases of established irreversible fibrosis. However, longitudinal observational studies on inflammatory markers in SSc, defining this inflammatory phase, are scarce.

C-reactive protein (CRP) is produced by hepatocytes upon stimulation by interleukin 6 (IL-6) and is an acute-phase reactant [5]. Cross-sectional studies demonstrated associations between CRP and disease activity, diffuse cutaneous SSc (dcSSc), the presence of anti-Scl-70 antibodies, modified Rodnan skin score (mRSS), pulmonary function, and reduced survival [6–13]. Since CRP assessments have become the standard of care, retrospective studies on its value as an inflammatory marker in SSc are feasible.

Data from a large retrospective study showed an association of elevated CRP levels at baseline (defined as > 8 mg/l) and short disease duration [6]. Data from another cohort investigating 266 patients (GENISOS) did not find an association between early disease and CRP levels [7]. Another study demonstrated increased CRP levels in established SSc compared with patients with undifferentiated connective tissue disease at risk for SSc [8]. Hence, data on elevated CRP levels in the early stages of the disease are contradictory.

Previous studies assessed cross-sectional CRP values only except for two studies. One study analyzed data on mean and median CRP levels over the course of six annual follow-up visits, which demonstrated no significant change in CRP levels over the observed time span [6]. Another study, with a high proportion of limited cutaneous SSc (lcSSc) patients, revealed that a twofold increase in CRP between visits is associated with a decrease in pulmonary function but not with mRSS [13]. In both studies, individual CRP courses were not depicted. Although occasions of CRP elevations were captured in the latter study, subgroups according to longitudinal CRP profiles were not analyzed and conclusions on inflammatory phases of the disease were not drawn.

In our own observation, some patients are characterized by long-standing elevation of CRP levels, whereas other patients never show CRP elevations.

Hence, this study has been designed to investigate CRP levels longitudinally in a cohort of SSc patients to evaluate the paradigm of an initial inflammatory phase of the disease (as defined by CRP elevation). The aim was to determine the (i) longitudinal CRP levels in individual patients; (ii) associations between persistent CRP elevations, disease characteristics, and comorbidities; and (iii) the impact of immunosuppressive therapies on CRP levels.

## Methods

### Study population

In this retrospective study, patients with SSc treated in the outpatient clinic of the Department of Rheumatology and Clinical Immunology, University Medical Center Freiburg, between 1997 and 2015, were evaluated. This study has been approved by the Ethics Committee of the University of Freiburg (ethic vote 290/17). Patients’ charts were reviewed retrospectively by one investigator and controlled by a second investigator using a pre-specified data assessment form. Patients with SSc according to the 2013 ACR/EULAR classification criteria, at least three consecutive visits and at least 1 year follow-up, were included [1]. CRP elevations related to infections or medical interventions, as evidenced by retrospective chart review, were excluded from the analysis. CRP serum levels were documented at every visit and categorized as positive if CRP concentrations were ≥ 5 mg/l. Subjects with elevated CRP levels at more than 80% of visits were defined as inflammatory SSc while patients with normal CRP levels on all visits were defined as non-inflammatory SSc.

### Clinical and laboratory parameters

Laboratory and clinical parameters were determined by chart review: CRP, anti-centromere antibody, anti-Scl-70 antibody, age, sex, body mass index (BMI), SSc subsets (dcSSc, lcSSc), disease duration, immunosuppressive treatment, prevalence of calcinosis cutis (according to physical examination or imaging), cardiac arrhythmia (based on diagnosis/medical letter and/or ECG), atherosclerosis (as demonstrated by ultrasound, radiography, magnetic resonance imaging, or computed tomography (CT)), coronary artery disease (as evidenced by left heart catheterization or CT), congestive heart failure, hypertension, malignancy, gastrointestinal or esophageal involvement (based on imaging, endoscopy, or clinical diagnosis), arthritis (as shown by ultrasound or clinical examination), rheumatoid arthritis (RA) (as evidenced by physician’s diagnosis and erosions and/or anti-CCP positivity), Sjögren’s syndrome (SjS), anemia (as defined by hemoglobin; male < 13 g/dl, female < 12 g/dl), pulmonary fibrosis (based on CT), pulmonary arterial hypertension (PAH) if confirmed by right heart catheterization (defined by mean pulmonary artery pressure at rest ≥ 25 mmHg), pulmonary function tests, and mRSS. If not specified otherwise, comorbidities were defined by diagnosis as documented in the medical letter. Disease duration was measured from the date of primary diagnosis until the respective assessments.

### Statistical analyses

All values are shown as median (range). The Mann-Whitney *U* test, Kruskal-Wallis test, and the chi-square test were used to compare the medians and proportions.

Correlations were analyzed using Spearman’s rank correlation coefficient. The Wilcoxon signed-rank test was used to determine the change in CRP levels before and after CYC treatment. All hypothesis tests were two-sided, and *p* values < 0.05 were considered statistically significant. Stata software (version 13.1, StataCorp, College Station, TX, USA) and SPSS (version 25, IBM Corp., New York, USA) were used for data management, statistical analysis, and graphing of the results.

## Results

### Total cohort

One hundred thirty-one SSc patients were included in this study with an observation time of 7.6 (1.0–19.5) years. A total of 1815 consecutive visits of 131 SSc patients were analyzed. Patients’ characteristics, number of visits, and observation time are shown in Table 1. Median disease duration at first visit was 0 (0–27.8) years. Sixty-five patients (50%) were diagnosed in the study center, and therefore, CRP levels of half of the patients were available from the date of primary diagnosis.

**Table 1:** Patients’ characteristics, listed for all patients, and inflammatory, non-inflammatory, and intermediate systemic sclerosis (SSc)

	All patients (*n* = 131)	Inflammatory (*n* = 24)	Non-inflammatory (*n* = 38)	Intermediate (*n* = 69)	*p* value^3^
Age [years]	54 (18–78)	60 (24–77)	50 (20–78)	55 (18–77)	0.041
Male (*n*/%)	24/18.3	9/38	4/11	11/16°	0.022
Body mass index [kg/m^2^]	24.3 (16.4–38.1)	25.4 (16.4–36.8)	23.0 (18.8–33.4)	25.6 (19.3–38.1)^#^	0.069
Disease duration [years]	0.0 (0–27.8)	0.4 (0.0–22.1)	0.0 (0.0–10.2)	0.3 (0.0–27.8)^#^	0.053
Number of visits per patient	13 (3–38)	13 (5–26)	11 (3–21)	16 (3–38)^#^	0.365
Observation time [years]	7.6 (1.0–19.5)	5.8 (1.0–16.1)	7.0 (1.9–15.8)	9.3 (1.1–19.5)^#^°	0.245
dcSSc/lcSSc (%)	42/58	67/33	37/63	36°/64°	0.036
Scl-70 *(n*/%)	53/40	16/67	12/32	25/36°	0.009
Anti-centromere (n/%)	59/45	4/17	23/61	32/46°	0.001
Anemia (*n*/%)	28/21	8/33	7/18	13/19	0.229
mRSS	10 (0–41)	18 (0–41)	7 (0–22)	10 (0–31)°	< 0.001
FVC (*n* = 101) [%]	92 (33–132)	76 (33–108)	102 (64–132)	92 (62–130)°	< 0.001
TLC (*n* = 100) [%]	85 (44–118)	76 (44–118)	92 (58–117)	86 (54–117)°	0.001
DLCO (*n* = 96) [%]	75 (26–121)	57 (28–101)	83 (39–121)	77 (26–101)	0.008
Pulmonary fibrosis (*n*/%)	60/46	20/83	11/29	29/42°	< 0.001
Calcinosis cutis (*n* = 130/%)	26/20	6/25	8/21	12/18	0.762
Arthritis (*n*/%)	26/20	6/25	8/21	12/17	0.762
Rheumatoid arthritis (*n* = 130/%)	10/8	5/21	0/0	5/7	0.007
Pulmonary hypertension^1^ (*n*/%)	25/19	6/25	7/18	12/17	0.541
Cardiac arrhythmia (*n* = 130/%)	18/14	6/25	2/5	10/14	0.048
Arterial hypertension (*n*/%)	55/42	13/54	13/34	29/42	0.186
Atherosclerosis (*n*/%)	16/12	4/17	1/3	11/16	0.069
Coronary artery disease (*n* = 130/%)	11/8	3/13	0/0	8/12^#^	0.056
Congestive heart failure (*n*/%)	3/2	2/8	0/0	1/1	0.146
Esophageal involvement (*n*/%)	90/69	17/71	25/66	48/70	0.784
Gastrointestinal involvement (*n* = 128/%)	19/15	2/8	3/8	14/21	1.000
Sjögren’s Syndrome (*n* = 127/%)	8/6	0/0	2/5	6/9	0.515
Malignancy (*n* = 128/%)	23/18	6/25	8/21	9/14	0.762
Prednisone (*n*/%)	51/39	15/63	9/24	27/39	0.003
Immunosuppressive treatment ever* (*n*/%)	72/55	19/79	14/37	39/57	0.002
Methotrexate^2^ (*n*/%)	33/25	8/33	4/11	21/30	0.046
Azathioprine^2^ (*n*/%)	19/15	3/13	3/8	13/19	0.669
Leflunomide^2^ (*n*/%)	9/7	3/13	2/5	4/6	0.366
Cyclosporine^2^ (*n*/%)	5/4	1/4	2/5	2/3	1.000
Mycophenolate mofetil^2^ (*n*/%)	38/29	10/42	8/21	20/29	0.094
Abatacept^2^ (*n*/%)	2/2	1/4	0/0	1/1	0.387
Rituximab^2^ (*n*/%)	15/11	5/21	2/5	8/12	0.098
Cyclophosphamide^2^ (*n*/%)	30/23	12/50	5/13	13/19°	0.007

Values are shown as median (range) or absolute numbers/percentages (*n* = 131 if not specified)

*CRP* C-reactive protein, *dcSSc* diffuse cutaneous SSc, *DLCO* diffusing capacity, *FVC* forced vital capacity, *lcSSc* limited cutaneous SSc, *mRSS* modified Rodnan skin score, *TLC* total lung capacity

*Immunosuppressive treatment including methotrexate, azathioprine, leflunomide, cyclosporine, mycophenolate mofetil, rituximab, abatacept, and cyclophosphamide

^#^*p* value ≤ 0.05 as determined by Mann-Whitney *U* test or chi-square test comparing intermediate and non-inflammatory patients

^°^*p* value ≤ 0.05 as determined by Mann-Whitney *U* test or chi-square test comparing intermediate and inflammatory patients

^1^Confirmed by right heart catheterization

^2^Number of patients who have ever received the listed treatment

^3^*p* values were determined by Mann-Whitney *U* test or chi-square test comparing inflammatory and non-inflammatory patients

There was no difference between CRP levels at first visit (3.4 (2.9–50.1) mg/l) vs. last visit (3.3 (1.3–88.6) mg/l) in the total cohort (*n* = 131, *p* = 0.250). Moreover, there was no association between disease duration or BMI and CRP levels at first visit (*p* = 0.118, *p* = 0.750). Smoking status was documented in 77.1% of the total population (*n* = 101) and 72.6% of patients with inflammatory or non-inflammatory SSc (*n* = 45). There was no difference in CRP level at the first visit between active smokers and non-smokers in the total cohort (*p* = 0.717). CRP levels at first visit did not differ between patients receiving methotrexate (*p* = 0.361) or CYC (*p* = 0.916) and those who did not.

### Inflammatory versus non-inflammatory SSc

**Fig. 1:**
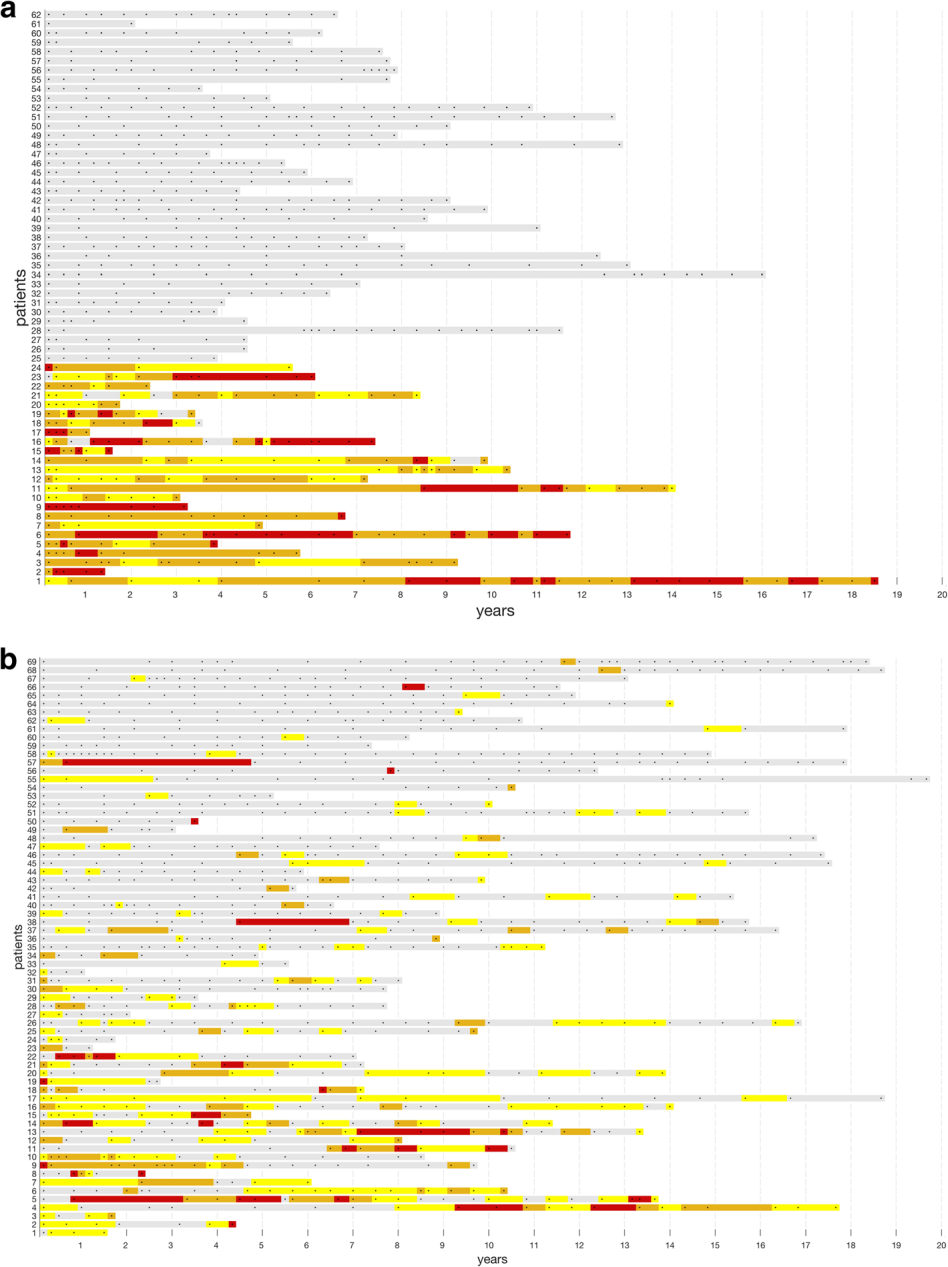
**a** C-reactive protein (CRP) levels of patients (*y*-axis) with inflammatory systemic sclerosis (SSc) (*n* = 24, patients 1–24) and non-inflammatory SSc (*n* = 38, patients 25–62) over the course of time. **b** CRP levels of the intermediate group (*n* = 69, patients 1–69) over the course of time. *x*-axis = years. Each dot in the line represents one visit. CRP < 5 mg/l, gray; CRP ≥ 5 to < 10 mg/l, yellow; CRP ≥ 10 to < 20 mg/l, orange; CRP > 20 mg/l, red

A total of 18.3% (*n* = 24) presented with inflammatory SSc as defined above, whereas in 29% (*n* = 38), CRP levels were always in the normal range (Fig. 1a).

**Fig. 2:**
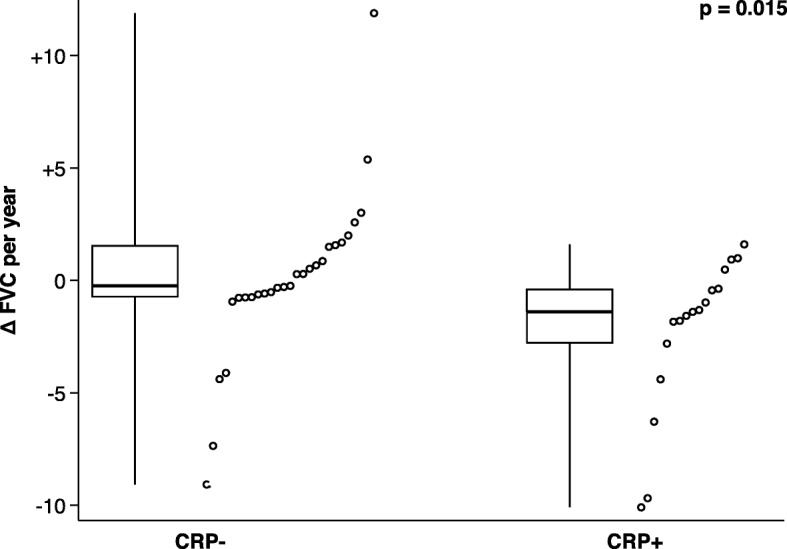
Decline of forced vital capacity (FVC) per year in the non-inflammatory (CRP−) compared to the inflammatory (CRP+) group. Dots represent individual FVC values (spaced out across abscissa for better visibility). Box plots represent the 25th to 75th percentile with the thick line denoting the median. Whiskers cover the entire data (minimum to maximum)

Patients with inflammatory SSc presented more frequently with older age (*p* = 0.041), male gender (*p* = 0.022), anti-Scl-70 antibodies (*p* = 0.009), dcSSc (*p* = 0.036), pulmonary fibrosis (*p* < 0.001), RA (*p* = 0.007), and cardiac arrhythmia (*p* = 0.048) as compared with non-inflammatory SSc. Moreover, patients with inflammatory SSc revealed higher mRSS (*p* < 0.001) and lower forced vital capacity (FVC) (*p* < 0.001), total lung capacity (TLC) (*p* = 0.001), and diffusing capacity (DLCO) (*p* = 0.008) in comparison with non-inflammatory SSc (Table 1). The decline of FVC per year was significantly greater in the inflammatory group compared to the non-inflammatory group (Fig. 2). Patients in the inflammatory group were more likely to receive immunosuppressive treatment compared to the non-inflammatory group (79 vs. 37%) (*p* = 0.002), including treatment with prednisone (63 vs. 24%) (*p* = 0.003), CYC (50 vs. 13%) (*p* = 0.003), and methotrexate (33 vs. 11%) (*p* = 0.046) (Table 1). The prevalence of smoking was not different between patients with inflammatory and non-inflammatory SSc (*p* = 0.331).

In the inflammatory subgroup, CRP levels were slightly higher at last visit 13.2 (3–88.6) mg/l compared to first visit 9.5 (3–50.1) mg/l (*p* = 0.017); no difference was found for non-inflammatory SSc (first visit, 3.2 (2.9–4.0) mg/l; last visit, 3.1 (2.9–4.8) mg/l; *p* = 0.342).

### Intermediate group

In 69 patients (52.7%), CRP was positive at < 80% of visits. The CRP courses of this intermediate group are shown in Fig. 1b.

**Fig. 3:**
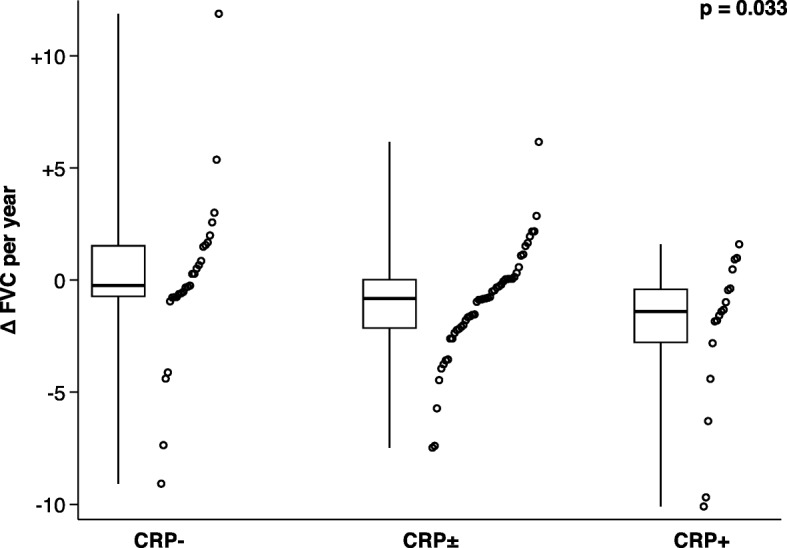
Decline of forced vital capacity (FVC) per year in the non-inflammatory (CRP−), intermediate (CRP±), and inflammatory (CRP+) groups. Dots represent individual FVC values (spaced out across abscissa for better visibility). Box plots represent the 25th to 75th percentile with the thick line denoting the median. Whiskers cover the entire data (minimum to maximum). The *p* value was calculated by Kruskal-Wallis test comparing all three subgroups

The intermediate group was characterized by longer disease duration (*p* = 0.035), longer observation time (*p* = 0.037), and more visits per patient (*p* = 0.011) as compared to the non-inflammatory group. Moreover, these patients showed higher BMI (*p* = 0.021), more coronary artery disease (*p* = 0.048), and a faster decline of FVC per year in comparison with non-inflammatory SSc (see Fig. 3). There was a trend towards more atherosclerosis and methotrexate therapy in the intermediate group in comparison with the non-inflammatory group, which did not reach statistical significance (*p* = 0.052, *p* = 0.050). When comparing the intermediate group with inflammatory SSc, the following differences were found: longer observation time (*p* = 0.014), lower mRSS (*p* = 0.002), less pulmonary fibrosis (*p* = 0.001), higher FVC (*p* = 0.002), higher TLC (*p* = 0.005), lower prevalence of anti-Scl-70 antibodies (*p* = 0.016), higher prevalence of anti-centromere antibodies (*p* = 0.014), higher proportion of lcSSc (*p* = 0.016), lower proportion of dcSSc (*p* = 0.016), and less male patients (*p* = 0.042). Moreover, patients from the intermediate group were less likely to receive CYC as compared to the inflammatory group (*p* = 0.006). There was no difference between CRP levels at first versus last visit in the intermediate group (first visit, 3.4 (3.0–45.5) mg/l; last visit, 3.5 (1.3–47.0) mg/l; *p* = 0.940). For further characteristics of the intermediate group, see Table 1.

### Cyclophosphamide treatment

**Fig. 4:**
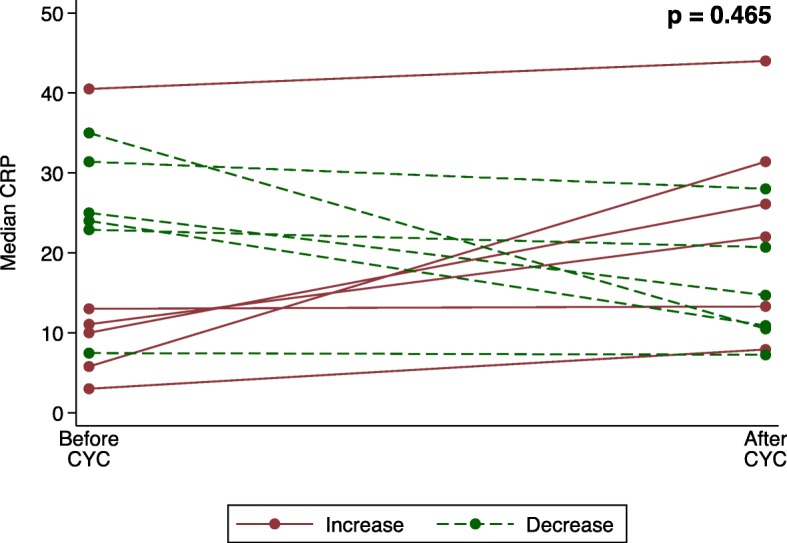
Median C-reactive protein (CRP) levels [mg/l] of 12 patients with inflammatory SSc before (18.0 (3.0–40.5) mg/l) and 1 year after (17.7 (7.3–44.0) mg/l) cyclophosphamide (*p* = 0.754)

**Fig. 5:**
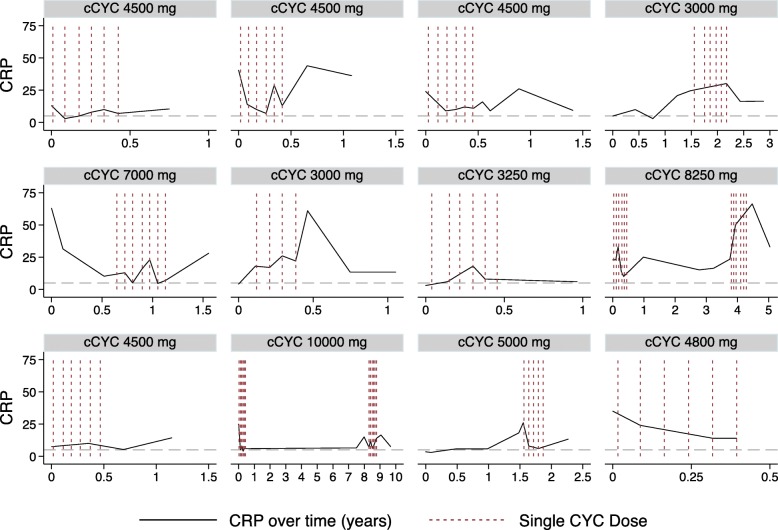
Individual C-reactive protein (CRP) courses of 12 patients with inflammatory systemic sclerosis (SSc) over time (years) receiving treatment with cyclophosphamide (CYC). Each vertical red line represents a single dose of CYC; horizontal dashed gray lines denote the CRP positivity threshold. cCYC refers to cumulative CYC dose of each patient. *y*-axis is the CRP level [mg/l]; *x*-axis is the time [years]

A total of 29 patients (23%) received treatment with CYC during the study period with a cumulative dose of 4.7 (3.0–40.5) g. There were no differences in CRP levels before and after treatment with CYC in the total cohort (4.7 (3.0–40.5) mg/l vs. 7.6 (3.1–44.0) mg/l; *p* = 0.239). Of all patients treated with CYC, 12 were classified as inflammatory SSc and 5 as non-inflammatory SSc. There were no differences in CRP levels before and after treatment in these subgroups (inflammatory, 18.0 (3.0–40.5) vs. 17.7 (7.3–44.0) mg/l, *p* = 0.754; non-inflammatory, 3.2 (3.0–3.2) vs. 3.3 (3.1–3.4) mg/l, *p* = 0.465; see Fig. 4). The individual CRP courses of the 12 patients with inflammatory SSc receiving treatment with CYC are shown in Fig. 5. Four of 5 patients with inflammatory SSc and concomitant RA were treated with CYC.

## Discussion

A subgroup of SSc patients shows persistent long-term CRP elevations and high morbidity. Even treatment with CYC did not alter CRP levels in these patients. Presumably, macrophages, less affected by CYC treatment, maintain inflammation in this subgroup. The individual analysis of CRP courses revealed two extreme subpopulations characterized by consistent CRP elevations (inflammatory SSc) or CRP values in the normal range (non-inflammatory SSc).

Parameters found to be more common in the inflammatory group, such as pulmonary fibrosis, cardiac arrhythmia, dcSSc, higher mRSS, reduced lung function, the presence of anti-Scl-70 antibodies, or higher age, are associated with increased mortality [14–18]. Moreover, the proportion of male gender, which has been linked to increased mortality in SSc [14, 15, 19], was higher in the inflammatory subgroup. No patient in the inflammatory group presented with SjS. An overlap with SjS has been shown to be associated with a rather mild SSc phenotype and lower frequency of lung fibrosis, PAH, or renal crisis [20].

Overall patients with inflammatory SSc seem to represent a subgroup with higher morbidity and mortality. Accordingly, patients with inflammatory SSc showed a faster decline of FVC over time and received more frequently immunosuppressive treatment (e.g., CYC).

Patients defined as the intermediate group (CRP positivity at < 80% of visits) revealed a phenotype in between inflammatory and non-inflammatory SSc. They showed a less severe phenotype as compared to inflammatory SSc, but a faster decline of FVC per year when compared to non-inflammatory SSc. Observation time, number of visits per patients, and disease duration were longer in the intermediate group compared to non-inflammatory SSc. This might be due to a decreasing probability of persistent CRP negativity during longer observation periods.

In our study, more than one third of patients (35.9%) had elevated CRP levels at baseline, which is higher compared to previous studies showing CRP positivity in approximately one quarter of patients (25.7 and 22.4%) [6, 7]. An Australian cohort demonstrated CRP elevations in 54% (not cross-sectional; including any CRP elevations during follow-up) and hence normal levels in 46%. This is higher than the 29% of non-inflammatory SSc observed in our cohort and might be due to the longer observation time of our study (mean 8.4 vs. 3.5 years) [13].

We observed no changes in the CRP levels over time, consistent with a previous study, which demonstrated stable mean CRP levels over six annual follow-up visits [6]. No correlation between disease duration and CRP levels (at baseline) was found, which is in line with the study published by Liu et al. (GENISOS cohort) [7], but stands in contrast to results from the Canadian Scleroderma Research Group, which demonstrated a correlation of baseline CRP levels with disease duration [6]. Mean disease duration in our study was 2.9 ± 5.6 years at baseline and therefore closer to the GENISOS (2.5 ± 1.6) than the Canadian (11.0 ± 9.5 years) cohort. We did not find any change in CRP levels over the observed time span, and CRP levels in the inflammatory subgroup were even slightly increased at last visit. Hence, discrepancies in studies on disease duration and CRP might rather be confounded by analyzing different phenotypes (inflammatory and non-inflammatory) than by leveling down of inflammation over time.

Our data demonstrates that CRP levels did not change even after treatment with CYC. This seems surprising, as CYC is one of the mainstays of SSc treatment, and theoretically, CRP levels should decline after such a potent B and T cell-targeted treatment. A previous study with 18 patients treated with oral CYC and prednisone demonstrated a decline in median CRP from 16 to 12 mg/l [21]. However, the decline was modest, all patients in this study were additionally treated with corticosteroids (67% in our study), and only single measurements of CRP levels before and 1 year after treatment were compared as opposed to our study measuring the median CRP levels before CYC and over 1 year after treatment. Although the analysis of clinical response to CYC was not possible in our study due to insufficient follow-up data and the retrospective design, a small study (*n* = 24) revealed that elevated baseline CRP levels are associated with poor response to CYC [22]. Presumably, other cell types, less affected by CYC, like macrophages, might explain these findings. Macrophages appear to be crucial players in orchestrating inflammation and fibrosis in SSc [23]. Inflammatory macrophages produce IL-6, thereby enhancing M2 macrophage differentiation [24]. CC chemokine ligand 18 (CCL18) produced by M2 macrophages stimulates collagen production in fibroblasts and is elevated in patients with lung fibrosis as well as in systemic sclerosis [25–27]. CCL18 was the only measured marker that significantly declined in SSc patients after treatment with tocilizumab (anti-IL-6) in a randomized controlled trial (FaSScinate) [23]. In contrast to tocilizumab, CYC primarily affects lymphocytes; hence, inflammatory macrophages might maintain high CRP levels and promote the progression of the disease in these patients with laboratory unresponsiveness to CYC.

We did not observe an association of CRP levels at first visit with BMI as opposed to other studies [6, 13]. Nevertheless, there were higher BMI values in the intermediate group and a not significant tendency towards higher BMI values in inflammatory SSc as compared to non-inflammatory SSc. Inflammation is a known risk factor for atherosclerosis [28]. Accordingly, we found a trend towards a higher prevalence of atherosclerosis and coronary artery disease in the intermediate group and in inflammatory SSc. Presumably, due to the small subgroups, this difference was only significant for coronary artery disease in the intermediate group as compared to non-inflammatory SSc.

Arthritis (of all causes) was not significantly associated with inflammatory SSc consistent with a previous study [6] but in contrast to the findings of EUSTAR and an Australian cohort [13, 29]. RA, in contrast, was associated with inflammatory SSc. However, only a total of ten patients in the cohort and five in the inflammatory group had an overlap with RA. Patients with RA/SSc seem to be characterized by a distinct genetic, clinical, and serological phenotype, and a high prevalence of pulmonary fibrosis (77%) [30], consistent with our study showing pulmonary fibrosis in 80% of SSc/RA patients (8/10). Moreover, in the inflammatory subgroup, four out of five patients with RA were treated with CYC. Both findings illustrate the severe phenotype of patients with inflammatory SSc.

Acknowledging the retrospective nature of this study, its strengths include the detailed characterization of the cohort and the long follow-up duration. Additionally, data for half of the patients were available from the time of primary diagnosis. Besides the retrospective study design, limitations include small sample sizes in subgroups and the definition of disease duration, which was measured from the diagnosis and not from the onset of first non-Raynaud’s symptom.

## Conclusions

Our study reveals a subgroup of SSc patients characterized by long-standing CRP elevations. Patients with such inflammatory SSc were characterized by high morbidity, including pulmonary fibrosis and cardiac arrhythmia, and a faster decline of pulmonary function. Even treatment with CYC did not alter CRP levels. These findings underscore the heterogeneity of the disease and emphasize the importance of defining subgroups in SSc, which potentially can guide treatment decisions. Whether patients with inflammatory SSc are more responsive to immunosuppressive treatment regimens has to be elucidated in further studies.

## Data Availability

The datasets used and/or analyzed during the current study are available from the corresponding author on reasonable request.

## Abbreviations

BMI: Body mass index
CCL18: CC chemokine ligand 18
CRP: C-reactive protein
CT: Computed tomography
CYC: Cyclophosphamide
dcSSc: Diffuse cutaneous systemic sclerosis
DLCO: Diffusing capacity
FVC: Forced vital capacity
HSCT: Hematopoietic stem cell transplantation
IL-6: Interleukin 6
ILD: Interstitial lung disease
lcSSc: Limited cutaneous systemic sclerosis
mRSS: Modified Rodnan skin score
PAH: Pulmonary arterial hypertension
RA: Rheumatoid arthritis
SjS: Sjögren’s syndrome
SSc: Systemic sclerosis
TLC: Total lung capacity
